# Current and Developing Therapeutics for Dry Eye Disease: Targeting Ion Channels

**DOI:** 10.3390/cimb48030332

**Published:** 2026-03-21

**Authors:** Rebecca Jung, Emily Kao, Victor H. Guaiquil, Ali R. Djalilian, Mark I. Rosenblatt

**Affiliations:** Illinois Eye and Ear Infirmary, Department of Ophthalmology and Visual Sciences, College of Medicine, University of Illinois at Chicago, Chicago, IL 60612, USA; rjung6@uic.edu (R.J.); vguaiqui@uic.edu (V.H.G.)

**Keywords:** dry eye disease, ion channels, neuropathic ocular pain, transient receptor potential channels, TRPM8, TRPV1, Nav channels, ocular surface

## Abstract

Dry eye disease (DED) is an ocular surface disorder characterized by tear film instability, inflammation, epithelial damage, and neurosensory abnormalities. Due to its multifactorial etiology and pathophysiology, conventional therapies that focus on lubrication and immunosuppression often fall short in addressing the neuropathic component of ocular pain experienced by a growing subset of patients. Recent developments in sensory neuroscience have highlighted the pivotal role of ion channels in mediating ocular surface homeostasis, pain signaling, and inflammation. This review examines the role of the following major ion channel families in the pathophysiology of DED and neuropathic ocular pain: transient receptor potential (TRP) channels, voltage-gated sodium (Nav) channels, and purinergic P2X receptors. The review details their anatomical distribution, molecular function, and responses to environmental stimuli such as heat, cold, osmolarity, and injury. Current treatments, such as artificial tears, anti-inflammatory drops, and systemic neuromodulators, are also reviewed in relation to their effects on ion channel modulation. Additionally, emerging therapies that directly target sensory transduction pathways are introduced. This review highlights the therapeutic potential of ion channel modulation in personalizing treatment for patients with ocular surface pain, particularly those with neuropathic features unresponsive to standard care.

## 1. Introduction

Dry eye disease (DED) is a common ocular surface disorder with a prevalence ranging from 5% to 30%, depending on the population, diagnostic criteria, age, sex, comorbidities, and environmental exposures [1,2]. These rates rise to 50% in older populations, making it a significant disease and socioeconomic burden worldwide [3], due to its chronicity that impacts visual function and decreases quality of life [4]. DED presents as a complex condition [5] characterized by clinical measures of decreased tear film and a wide range of symptoms, including burning, stinging, foreign body sensation, grittiness, photophobia, and poor vision [6]. Patients also report that these visual deficits significantly interfere with everyday tasks such as reading, driving, computer work, and outdoor activities, highlighting the need to address DED as a significant disease that affects quality of life [6,7,8].

The pathophysiology of DED has been studied from a multifaceted approach and involves many interrelated factors [3,9,10]. The Tear Film and Ocular Surface Society Dry Eye Workshop (TFOS DEWS) II currently defines DED as a disease caused by the loss of tear film homeostasis, resulting in tear film instability, increased tear osmolarity, ocular surface inflammation, epithelial damage, and neurosensory abnormalities [1]. While ocular damage and inflammation are most often linked to DED symptoms and presentation, a growing body of literature also cites neurosensory dysfunction and dysregulation as central disease effects [11,12,13] in a large subset of patients.

**Scheme 1:**
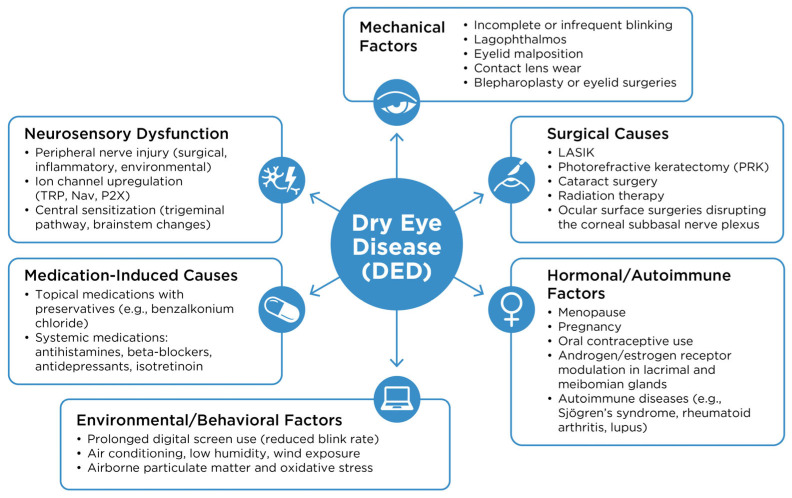
Schematic of the main identifiable etiological factors that contribute to DED.

As such, DED is not a singularly describable syndrome but rather has diverse underlying causes and a complex pathophysiology (Scheme 1). Tear film instability and hyperosmolarity are the most common and central mechanisms by which DED occurs [14,15]; however, other distinct mechanisms specific to different patient populations also exist. These causes can be categorized into mechanical, surgical, hormonal, environmental, medication-induced, and neurosensory dysfunction.

Mechanical causes include incomplete or infrequent blinking, lagophthalmos, and lid malposition [16,17]. All of these can lead to increased evaporative loss and poor tear film stability. Prolonged digital device use has also been associated with reduced blink rate, contributing to evaporative stress [18,19].

Surgical treatments, such as laser-assisted in situ keratomileusis (LASIK), photorefractive keratectomy (PRK), and cataract surgery, are highly attributed to inducing and/or exacerbating DED [20,21,22,23,24]. These procedures inherently need to damage the cornea’s epithelial layer, where the corneal subbasal nerve plexus is embedded. Disruption of the subbasal nerve plexus disrupts the reflex tearing and blink reflexes [25,26]. Postsurgical dry eye has been reported to persist for months to years due to chronic alterations in nerve morphology and function [21,22]. Other ocular surface surgeries, radiation therapy, and blepharoplasty may also cause surgically induced mechanical insults to the ocular surface, resulting in neural dysfunction [27].

Hormonal influences are another important contributor to dry eye pathophysiology, particularly in women [28,29,30]. Lacrimal and meibomian glands express estrogen and androgen receptors [31]. As such, hormonal fluctuations that occur with menopause, pregnancy, or use of oral contraceptives have been associated with increased DED prevalence in women [32,33]. Additionally, systemic autoimmune diseases such as Sjögren’s syndrome, rheumatoid arthritis, and systemic lupus erythematosus play prominent roles in DED, largely through inflammatory infiltration of the lacrimal glands and disruption of tear production [34,35].

Environmental factors also play a role in DED etiology. Constant exposure to air conditioning or climates with high wind, low humidity, and particulate matter can exacerbate DED symptoms by increasing tear evaporation and inducing oxidative stress [36,37]. Contact lens wearers are more likely to experience DED symptoms due to mechanical friction, reduced oxygen permeability, and disruption of the tear film lipid layer [38,39].

Certain medications are increasingly recognized as contributing factors to DED symptoms [40]. Topical medications that contain preservatives, such as benzalkonium chloride, can cause epithelial toxicity [41,42,43], while systemic drugs such as antihistamines, beta-blockers, antidepressants, and isotretinoin may also reduce overall tear production or alter tear composition [44,45].

More recently, sensory dysfunction and neurosensory abnormalities have been studied as core contributors to the symptoms and pathophysiology of DED [1]. Certain patients report severe discomfort and pain despite minimal or no clinical signs, indicating that inflammation or tear instability cannot fully account for their symptoms [46,47,48]. Instead, peripheral sensitization of corneal nociceptor ion channels and central sensitization along the trigeminal pain pathways can lead to amplified normal sensory inputs that are interpreted as pain signals [49,50]. Whether caused by surgery, inflammation, or environmental insult, peripheral nerve injury can lead to the upregulation of nociceptive ion channels and irregular spontaneous activity [51,52]. Central changes, such as increased excitability of second-order neurons and altered descending inhibition, contribute to the persistence of neuropathic pain even in the absence of ocular surface damage.

Current traditional therapies for DED include artificial tears, corticosteroids, cyclosporine, lifitegrast, and punctal plugs [9,53,54,55]. Although effective in addressing tear instability and inflammation, they are often insufficient for patients with neuropathic pain features and fail to address the sensory dysfunction behind the symptoms [56].

Growing evidence from the fields of ocular neuroscience and molecular biology has characterized ion channels as central regulators of corneal sensation, epithelial homeostasis, inflammation, and pain perception [57,58]. Ion channels such as transient receptor potential (TRP) channels [59], voltage-gated sodium channels (Nav) [60], and purinergic P2X receptors [61] are expressed on the epithelial surface and sensory neurons of the cornea. These channels detect stimuli such as temperature changes, osmotic shifts, mechanical forces, chemical irritants, and tissue damage and transmit subsequent electrical signals to the central nervous system. Dysregulation of these channels, resulting from an injury to the corneal epithelial surface and sensory neurons, can then lead to sensory dysfunction characterized by sensory hyperexcitability, aberrant pain, and abnormal tear reflexes [13].

As ion channels have been identified to have a pivotal role in sensory processing, they present as possible therapeutic targets for addressing the inflammatory and neuropathic mechanisms of DED. Development of pharmacological modulation of these channels holds dual potential: alleviation of pain and restoration of physiological sensory function and epithelial integrity. As such, numerous preclinical and clinical studies are underway that aim to fully elucidate the potential of ion channel modulators, including TRPM8 agonists, TRPV1 antagonists, Nav inhibitors, and P2X3 blockers [15].

Recent review papers have summarized the expression and physiological roles of ion channels in corneal homeostasis. However, the growing understanding of sensory neuron dysfunction and neuropathic pain in dry eye disease (DED) elicits the need for an updated, more clinically integrated understanding of ion channels in the current therapeutic landscape. In contrast to earlier reviews that primarily catalog ion channel expression and physiological function, this review paper focuses on how ion channels specifically contribute to the sensory and neuropathic components of DED, as well as how current treatments contribute to ion channel modulation. The paper will first outline an understanding of the differing etiologies of dry eye disease, with an emphasis on neuropathic pain mechanisms that underlie persistent ocular discomfort despite conventional lubrication and anti-inflammatory therapies. We will then review existing clinical treatments within the context of these distinct pathophysiologic mechanisms. Finally, recent advances in ion-channel-targeted therapeutics will be highlighted in the context of how they address neuropathic drivers of ocular surface pain.

## 2. Pathophysiology

### 2.1. Tear Film Instability and Hyperosmolarity

The pathophysiology of dry eye disease (DED) can be attributed to a multitude of factors, but at the core of all types of DED, whether evaporative (EDE) or aqueous-deficient (ADDE), are tear film instability and hyperosmolarity, serving as central events that drive a cascade of self-perpetuating mechanisms [2]. The ocular surface is constantly exposed to the risk of desiccation via evaporation that is usually remediated by efferent autonomic nerves to the lacrimal gland that modulate tear secretion and distribution in response to ocular surface signals from afferent sensory nerves [62]. When tear secretion is deficient in either quantity or quality, tear film hyperosmolarity can occur, which creates an inflammatory environment by initiating MAP kinase and NFκB signaling pathways in conjunction with the generation of inflammatory cytokines (IL-1) and the recruitment of inflammatory cell [14,15,63]. These conditions favor corneal and conjunctival epithelial and goblet cell apoptosis, damaging the ocular surface, and can eventually lead to tear film breakup, which tends to exacerbate tear hyperosmolarity and therefore completes a vicious cycle of events that lead to further ocular surface damage and the development of DED symptoms like discomfort and increased blink rate [64,65]. These symptoms are estimated to occur when the ocular surface reaches a concentration of 450 mOsm/L; however, threshold values for diagnosis have varied from 308 mOsm/L to 316 mOsm/L, with the wide range attributed to local fluctuations in the tear film that can induce hyperosmolarity hotspots [64]. Tear film hyperosmolarity is considered a hallmark of DED due to its involvement in the disease cycle; however, several other mechanisms play a key role in initiating the loss of tear film homeostasis that leads to the central hyperosmolarity event [65].

### 2.2. Meibomian Gland Dysfunction

One such mechanism is dysfunction of the Meibomian glands, which are modified sebaceous holocrine glands whose acini contribute meibomian lipid (meibum) to tear composition [66]. Meibum is composed mainly of nonpolar lipids such as wax and cholesterol esters and is delivered into a shallow reservoir on the skin of the lid margin just anterior to the mucocutaneous junction. Secreted in a liquid form, the meibum is spread onto the preocular tear film with each blink, stabilizing the tear film and protecting the ocular surface from abrasives and irritants [16,66]. The mechanism of Meibomian gland dysfunction (MGD) most relevant to EDE is hyposecretion, characterized by gland obstruction, often caused by the underlying pathophysiology of epithelial hyperkeratinization, which leads to acinar atrophy and gland dropout [16]. Aging, environmental stress, and stem cell renewal all play crucial roles in meibocyte health, leading to potential changes in lipid profile, inflammatory cell infiltration, meibocyte proliferation and differentiation, and gland size. The protective film formed by meibum secretion is essential for preventing excessive water evaporation, which is one of the driving factors of DED [66].

### 2.3. Internal Factors: Hormonal and Autoimmune

Other internal factors can influence DED pathophysiology by acting on multiple mechanisms. For example, hormonal effects on DED have been identified, with one such mechanism of action being testosterone’s upregulation of mRNAs for sterol regulatory element binding proteins and acetyl-CoA synthase, which are critical for the initiation of lipogenesis and are therefore implicated in the induction of meibomian lipids [67]. The gender differences in DED have also been confirmed at the population level. A large epidemiologic study has shown that women may have higher DED incidence. One study reports that among individuals aged 50 years and older, the prevalence of DED was 15.9% in women compared to 7.0% in men—more than twice as high in women as in men [68]. In another cross-sectional survey study, it was found that the overall U.S. adult women group (over 18 years of age) had an 8.8% DED prevalence, compared to a 4.5% DED prevalence in men [69]. This disparity can be attributed to the roles of estrogen and progesterone in suppressing lipid production, upregulating pro-inflammatory cytokines, and inducing lacrimal gland regression [28,29,30,31]. Insulin is another hormone that may affect DED symptoms, working synergistically with androgens to enhance the secretory function of the lacrimal gland [28,70].

In addition to hormonal changes that affect DED via lipid control mechanisms, autoimmune disorders may also initiate and perpetuate the inflammatory invasion of the ocular environment. The ocular mucosal tissues host a diverse microbiome that is regulated by immune cells on the ocular surface, which work in conjunction with the antimicrobial and immunoregulatory properties of tears [71]. CD4^+^ T cells have been shown to play the most prominent role in the immunopathogenesis of chronic dry eye, with activated CD4^+^ T cells sufficient to induce dry eye in mice [72]. As a result, in disorders like primary Sjögren’s syndrome, which is characterized by lymphocytic infiltration of exocrine glands and other organs, dry eye is one of the primary manifested symptoms [73].

### 2.4. External Factors: Iatrogenic and Environmental

Iatrogenic and mechanical factors can both initiate and exacerbate the pathogenesis of DED. Systemic drug usage is a common iatrogenic factor for DED; some, like chloroquine and ibuprofen, increase tear film evaporation and mechanical irritation due to deposition of drug crystals in tears [74]. In terms of surgical interventions, one of the most frequently performed corneal refractive surgeries, LASIK, can cause dry eye symptoms in up to 95% of patients, with a further subset developing chronic, severe dry eye beyond the immediate post-operative period [75]. The leading potential mechanism for LASIK’s contribution to dry eye pathophysiology is iatrogenic corneal nerve damage via disruption of the sub-basal nerve plexus and stromal corneal nerves during laser ablation of the cornea [75,76,77,78]. Other mechanisms include damage to conjunctival goblet cells during surgical suction [79]. Post-LASIK, inflammation near nerve endings may directly stimulate pain or exacerbate pre-existing dry eye by initiating a cytokine cascade that destabilizes the tear film and perpetuates ocular surface inflammation. Furthermore, the change in resulting corneal shape itself may alter tear distribution during blinking, thereby contributing to further instability of the tear film. In addition to ensuring the mixing and spread of tear film over the ocular surface, blinking also plays a crucial role in inhibiting aqueous tear evaporation and fueling the lacrimal pump. Previous studies have demonstrated a correlation between diminished blink rate, whether because of high mental load or neurological pathologies like Parkinson’s disease, and DED symptom severity. One such study found that for every 10% increase in the proportion of incomplete blinks, the odds of dry eye disease increased by 12% [80]. Furthermore, external environmental conditions can also augment the risk of DED [81]. There is evidence that extreme temperatures, low humidity, air pollution, low atmospheric pressure, and airborne allergens each serve as DED risk factors, primarily by increasing tear evaporation or driving central loss of tear film stability [1].

### 2.5. Neuropathic Mechanisms

While a multitude of factors may contribute to the hallmarks of DED that correspond to tear dysfunction, lesser studied is the class of dry eye patients who describe symptoms of neuropathic origin. The lacrimal gland is innervated by both the parasympathetic and sympathetic nervous systems, with stimulation occurring through neural reflexes from the ocular surface. Corneal nociception is uniquely sensitive, with one study estimating the density of nociceptors to be 300 to 600 times that of the dermis [82]. In addition to nociceptors, corneal sensory receptors include cold-sensing thermoreceptors, mechanoreceptors, and polymodal receptors that all contribute to the maintenance of ocular surface homeostasis [83]. Neuropathic ocular pain may result from damage via injury or inflammation of these peripheral or central nervous system corneal and conjunctival somatosensory nerves [52]. Resulting neuroplastic responses can then induce sensitization of corneal nerves via inflammatory mediators, which may become chronic and cause pain that is unresponsive to standard DED treatments [47]. Ion channels represent a main mechanism by which this perception, regulation, and possible sensitization may occur, and will be the focus of this review moving forward.

## 3. Ion Channels in Corneal Sensory Transduction

**Figure 1:**
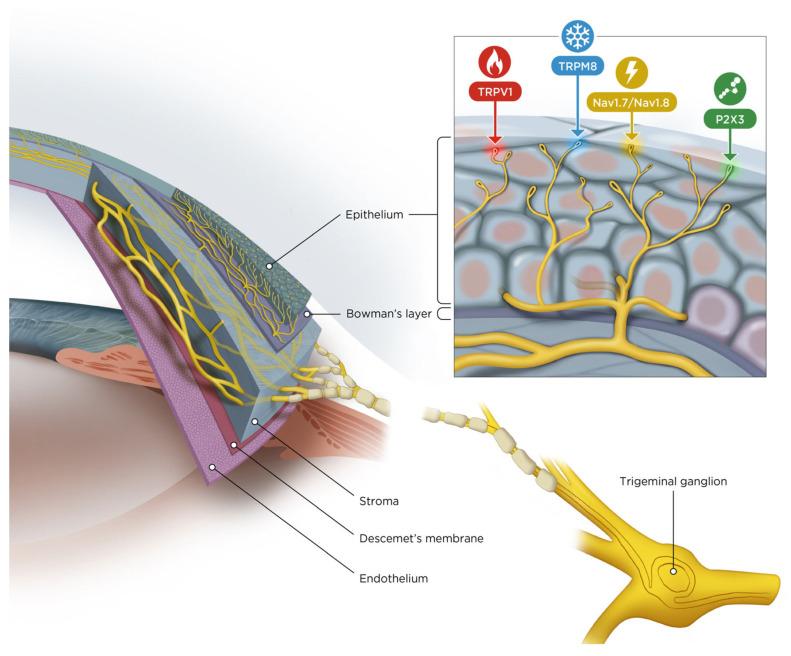
Layers of the cornea and nerves derived from the trigeminal ganglion entering the stroma and the different ion channel modalities expressed by the corneal nerves embedded in the corneal epithelium.

Corneal sensory transduction involves four major ion channel families that differ in their ability to transduce physical, thermal, or chemical stimuli to sensory neurons innervating the ocular surface (Figure 1).

### 3.1. Transient Receptor Potential (TRP) Family

The first of these is the Transient Receptor Potential (TRP) family, which comprises a diverse group of ion channels that aid sensory perception and are particularly critical for nociception, temperature sensation, and osmoregulation. Some TRP ion channels are partially voltage-dependent, like transient receptor potential melastatin 8 (TRPM8), while others are voltage-independent, like transient receptor potential ankyrin 1 (TRPA1), but all maintain a role in monitoring cellular ion levels and therefore are crucial for regulating the health of the ocular surface [59]. The distribution of specific TRP ion channels varies across the depth and cell types of the cornea. Transient receptor potential vanilloid 1 (TRPV1) and TRPA1 are predominantly at corneal nerve endings and have a role in corneal pain response in dry or inflammatory conditions. TRPM8 is primarily expressed in corneal nerve endings and corneal epithelial cells, playing a crucial role in cold sensation by facilitating enhanced responses to temperature changes. Transient receptor potential cation channel subfamily V member 4 (TRPV4) ion channels are distributed in epithelial, goblet, and immune cells. They are implicated in the transmission of mechanosensations as well as in the regulation of corneal injury and inflammation [84]. Due to their distinct structural properties and crucial roles in sensory transduction, TRP channels serve as attractive targets for drug development [85].

### 3.2. Voltage-Gated Sodium Ion Channels (Nav)

Another family of ion channels important to corneal sensory transduction is the voltage-gated sodium ion channel family (Nav), which serves as an important mediator of electrical signaling [60,86,87]. They are activated by depolarization of the neuronal membrane, changing their conformation to allow an inward current of positive sodium ions and thereby generating the ascending phase of an action potential. Because of their involvement in this integral step in transduction, Nav ion channels have often been studied in relation to crosstalk with other ion channels [86]. For example, blocking the activity of TRPM8 and TRPV1 receptors has been shown to decrease mRNA expression of Nav proteins [57,84]. Nav channels are often modulated by the addition of charged groups to intracellular, extracellular, or transmembrane domains, which alter the function of their gates and their interactions with other regulatory proteins at binding sites. Inflammation is often involved in post-translational modification of Nav ion channels via the activation of various intracellular kinases that alter their function or expression at the cell membrane [88]. Alterations in homeostatic Nav channel function or expression may lead to increased sodium influx and, therefore, neuronal hyperexcitability, which may be implicated in the pathogenesis of neuropathic pain [87,88].

### 3.3. Purinergic Receptor (P2X) Family

Purinergic receptors (P2X) are a family of ligand-gated, non-selective cationic ion channels that are permeable to sodium, potassium, and calcium ions, and often play a role in bridging inflammatory responses with ocular surface nociception [57,89]. A majority of P2X receptor types are expressed mainly in the trigeminal ganglia, but P2X7 receptors have been identified in the corneal epithelium and, along with P2X3, in the lacrimal gland [89,90]. P2X receptors are activated by extracellular ATP, which is commonly released due to tissue injury and inflammation, leading to the further release of inflammatory cytokines and the amplification of the inflammatory response. P2X activity is modulated allosterically by protons, with acidification playing a role in both the potency of agonists and direct inhibition. Immune cells at sites of inflammation often exhibit increased expression of P2X ion channels, making them a promising target for inhibiting the inflammatory response [91]. 

## 4. Ion Channels Contributing to Corneal Sensory Dysfunction in Dry Eye Disease

Within the TRP family of ion channels involved in corneal sensory transduction, three subtypes have been identified as playing a key role in the pathogenesis and perpetuation of DED and neuropathic ocular pain. 

### 4.1. TRPV1—Inflammation, Heat, and Capsaicin

The TRPV1 subfamily of receptors, commonly referred to as the capsaicin receptor, remains one of the most extensively studied TRP channels due to its critical role in maintaining tissue homeostasis and initiating the corneal wound-healing response [57,58,59]. It is a non-selective cation channel that serves as a multimodal nociceptor triggered by a variety of stimuli, including inflammatory mediators, acidity, heat above 43 °C, hyperosmolarity, and its main agonist, capsaicin [92,93,94]. The mechanism by which TRPV1 produces biological effects is via the mediation of extracellular cation (Ca^2+^, Na^+^, Mg^2+^) influx in both excitable and non-excitable cells, allowing for the conversion of physical and chemical stimuli to cellular signals in a distinctly non-overlapping fashion [95]. Capsaicin- and hydrogen-induced responses rely on calcium signaling, whereas thermal stimulation is calcium-independent [96,97]. Present in the corneal endothelium, epithelium, nerve fibers, conjunctiva, and trigeminal ganglion, TRPV1 is crucial for injury sensation and ocular surface inflammation. One function of TRPV1 is as a hypertonic osmotic sensor, and inhibition of TRPV1 may therefore provide protection against ocular surface damage that results from hyperosmolarity by preventing Ca^2+^ influx. The reduction in tear production associated with DED can lead to noxious stimuli that sensitize TRPV1 receptors, thereby enhancing the upregulation of intracellular responses linked to inflammatory and neuropathic pain [98]. Such responses include the activation of mitogen-activated protein kinase (MAPK) and nuclear factor κB (NF-κB), which increase the production of pro-inflammatory cytokines and recruit mature dendritic cells [99]. In contrast, increased TRPV1 expression may also activate neuroprotective systems after long-term injury, as demonstrated in a mouse model following excision of the lacrimal gland. The capsaicin pathway, which activates epidermal growth factor receptors (EGFRs), and MAPK signals, can also promote the proliferation and migration of epithelial cells to areas of injury. Inhibition of TRPV1 has been shown to reduce mucin and goblet cells, induce apoptosis of corneal epithelial cells, and decrease ocular surface moisture [98].

### 4.2. TRPM8—Cold Sensing and Basal Tear Regulation

In conjunction with TRPV1’s role in temperature-induced pain sensation, TRPM8 receptors are essential for cold sensing at thresholds of approximately 25 °C and exhibit responsiveness to cooling agents such as menthol and icilin [100,101]. Present in the corneal epithelium, endothelium, and eyelid, TRPM8 receptors are activated on the ocular surface after evaporation of the tear film induces changes in temperature and osmolarity, exhibiting tonic, spontaneous activity. As a result, they play an important role in monitoring eye wetness, and decreased TRPM8 expression has been shown to reduce basal tear secretion. On the other hand, hyperactivity of these cold thermoreceptors on the cornea has also been identified as a risk factor for abnormal lacrimation and, therefore, DED, which may result from increased irritation and cold hypersensitivity of the ocular surface [58,100,102,103]. The use of cooling as a treatment for ocular pain has been contested; however, the success of ice packs and cooling eye drops in relieving orbital pain and post-cataract surgery pain, respectively, provides evidence for the potential benefits of TRPM8 as a therapeutic target [57]. Furthermore, TRMV1 and TRPM8 interactions have been implicated in DED, as TRPV1 has been shown to hypersensitize the response of TRPM8-expressing neurons to cold discomfort, and this communication channel could also serve as a potential therapeutic target [104].

### 4.3. TRPA1—Oxidative and Chemical Irritants

An additional TRP ion channel with key relationships to DED and ocular pain is TRPA1, which is expressed in corneal sensory nerves, epithelial, endothelial, stromal, and immune cells, playing a similar yet significant role in pain reception via transduction of mechanical signals and a partial role in cold temperature sensation [59,105,106]. In addition to activation by inflammatory mediators, TRPA1 has been shown to respond to oxidative stress and environmental irritants, such as acrolein and allyl isothiocyanate [107]. In vitro studies have demonstrated TRPA1’s sensitivity to carbon dioxide due to its contribution to increased acidity [106]. The role of TRPA1 receptors in the pathogenesis of DED is hypothesized to result from increased expression in the trigeminal ganglion, which, upon activation, further releases pro-inflammatory neuropeptides, leading to the hallmark neurogenic inflammation and ocular pain [108]. TRPA1 antagonists have been demonstrated to reverse mechanical hypersensitivity and ocular discomfort. In contrast to TRPV1, their inhibition has been hypothesized to promote corneal nerve regeneration by reducing neuropeptide mediators of inflammation [105].

### 4.4. Nav1.7/1.8—Action Potential Generation and Ectopic Firing

In addition to the TRP family of ion channels, Nav1.7 and 1.8, both members of the class of voltage-gated sodium ion channels that play a crucial role in mediating electrical signaling in neurons, also contribute to ocular sensation and pain [109,110]. While their direct contribution to DED’s pathophysiology has not yet been established, both channels are relevant to ocular surface health due to their relationship with other ion channels and their activity. For example, blocking TRPM8 and TRPV1 activity has been shown to reduce mRNA expression of Nav1.7 and Nav1.8 in DED animal models compared to controls [109,110]. In contrast, it has been demonstrated that inflammation-driven post-translational modifications of these Nav ion channels, resulting from DED conditions, enhance the function or expression of Nav1.7/1.8, leading to increased sodium influx and, therefore, nociceptive neuronal hyperexcitability that may contribute to ectopic firing [57,110].

### 4.5. P2X3–ATP Signaling and Neurogenic Inflammation

P2X3 channels also emerge as potential contributors to DED-associated ocular pain, as they are strongly expressed in small trigeminal sensory neurons and exhibit the most rapid ATP-induced desensitization among the purinergic receptor family [90,111]. While there is a lack of specific studies on P2X3 receptors in the sensory nerve endings of the anterior eye, they represent a potential mechanism for DED pathogenesis due to their localization in the lacrimal gland [90]. Like other purinergic receptors, the stimulation of P2X3 ion channels by extracellular ATP released during tissue injury and inflammation implicates them in exacerbating the inflammatory response central to DED [112]. Interestingly, P2X3 has been shown to respond to hormonal regulation, with estrogen exerting inhibitory, anti-nociceptive effects that exert the opposite effect on DED symptoms compared to its regulation of lipid secretion [113].

## 5. Current DED Treatments and Their Relation to Ion Channels

**Figure 2:**
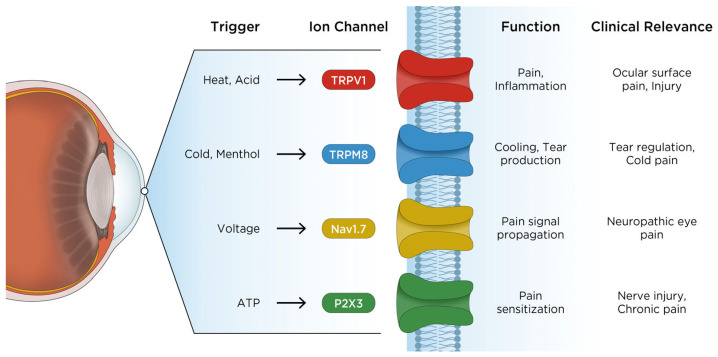
Ion channels expressed in the corneal nerves are depicted with their associated stimuli and related clinical relevance.

The therapeutic landscape for DED and neuropathic ocular pain has undergone significant evolution and development over the past two decades [54]. Management has traditionally been based on symptom control, ranging from simple lubricant-based regimens to targeted anti-inflammatory and neuromodulatory strategies [54]. However, novel understanding of the mechanistic role of ion channels in the pathogenesis of DED and neuropathic ocular pain (Figure 2) warrants re-evaluation of existing therapies and how they address—or fail to address—critical molecular pathways underlying the disease [58]. There may be indirect and/or unintended modulatory effects on ion channels, highlighting the potential for optimized treatments that fully leverage these molecular mechanisms [58].

### 5.1. Artificial Tears and Tear Film Stabilizers

Artificial tears and tear film stabilizers are the first-line treatment for DED patients [114]. These therapeutics are formulated with varying viscosities, osmolarities, and compositions, aiming to restore the volume and stability of the tear film, thereby reducing osmolar stress and improving ocular comfort [115]. While artificial tears do not directly modulate ion channels, their use may have indirect modulatory effects by diluting pro-inflammatory mediators and reducing osmotic and mechanical stimuli that trigger nociceptive ion channels [116].

For example, TRPV1 and TRPA1 channels are highly sensitive to dry, inflamed corneal environments and stimuli, such as hyperosmolarity, heat, and oxidative stress [117]. With the addition of artificial tears, tear film osmolarity and mechanical shear stress are decreased, resulting in reduced stimuli that would normally trigger TRPV1 and TRPA1 [65,92,118].

Newer formulations may also contain osmoprotective agents, such as trehalose or erythritol, which stabilize cellular osmotic balance and may reduce the activation of osmolarity-sensitive ion channels [65,115,119]. Lipid-based artificial tears help stabilize the outer lipid layer of the tear film, subsequently reducing evaporative loss and protecting cold-sensitive TRPM8-expressing neurons from inappropriate activation due to increased evaporative cooling [120,121].

### 5.2. Anti-Inflammatory Agents: Cyclosporine, Lifitegrast, and Corticosteroids

The use of anti-inflammatory agents, such as cyclosporine A, lifitegrast (Xiidra), and corticosteroids, has increased as chronic inflammation is recognized as a key driver of DED. By reducing immune cell activation and suppressing the production of pro-inflammatory cytokines, these therapies enhance tear production and ocular surface integrity [122,123,124,125].

Cyclosporine A (e.g., Restasis, Cequa) specifically inhibits calcineurin. Calcineurin is a phosphatase essential for T-cell activation, and its inhibition reduces IL-2 production and T-cell proliferation [122,123]. Conversely, lifitegrast antagonizes lymphocyte function-associated antigen 1 (LFA-1) by blocking its interaction with intercellular adhesion molecule 1 (ICAM-1), thereby preventing immune cell trafficking to the ocular surface [124]. Corticosteroids exert a broader suppression of inflammatory gene expression through glucocorticoid receptor-mediated transcriptional repression [126].

These agents can indirectly reduce the expression and sensitization of ion channels involved in nociceptive signaling [126,127]. Pro-inflammatory cytokines such as IL-1β, TNF-α, and IL-6 are known to upregulate TRPV1, TRPA1, and Nav1.7 channels in peripheral neurons. However, via agent-mediated attenuation of the inflammatory milieu, there may be a reversal of sensitization of corneal afferents and reduced aberrant ion channel activation [106,128]. There are also active investigative studies that establish a correlation between corticosteroid use and the transcriptional regulation of ion channels through nuclear hormone receptor interactions with the TRPV1 promoter region [127,129,130].

### 5.3. Punctal Occlusion: Mechanical Tear Retention and Indirect Sensory Modulation

Punctal plugs are most commonly used in moderate to severe cases of DED and aim to enhance tear retention by mechanically obstructing tear outflow through the lacrimal drainage system [131,132,133]. Though primarily mechanical, punctal occlusion may have additional downstream molecular effects that may influence ion channel activity. By increasing the retention time of the tear film, punctal plugs can also dilute surface pro-inflammatory mediators [134,135]. Similar to artificial tears and tear film stabilizer, this dilution mechanism helps reduce osmotic and desiccative stress, which may potentially normalize sensory neuron activation thresholds.

Punctal occlusion has notably varying clinical outcomes. Some patients report paradoxical exacerbation of ocular discomfort. This could be attributed to the accumulation of inflammatory cytokines or to disrupted sensory feedback loops due to altered tear dynamics [132,133]. More recent studies suggest that patients who present with the neuropathic pain phenotype with symptoms of persistent dysesthesia, allodynia, or spontaneous pain may be more susceptible to exacerbated symptoms after occlusion due to a lack of tear flow modulation and flushing of sensitized ion channels [134]. These clinical presentations indicate a need to phenotype patients before implementing tear occlusion [48,134].

### 5.4. Neuromodulatory Agents: Targeting Ion Channels in Ocular Pain

In clinical cases where neuropathic pain predominates, systemic or topical neuromodulators are next-line treatment options [48,134,136]. These include gabapentin, pregabalin, tricyclic antidepressants (TCAs), serotonin–norepinephrine reuptake inhibitors (SNRIs), and sodium channel blockers [48].

Gabapentin is not classically used for ocular applications; however, it has been found that low-dose systemic gabapentin can be applicable for treating chronic ocular pain syndromes that are treatment-resistant to topical therapy. Gabapentinoids bind to the α2δ subunit of voltage-gated calcium channels (VGCCs), thereby reducing calcium influx and subsequently inhibiting the release of excitatory neurotransmitters, such as glutamate and substance P [137,138]. TCAs and SNRIs inhibit sodium and potassium channels in nociceptive pathways, acting via both central mechanisms and local effects on ion channel function [139].

Topical sodium channel blocker anesthetics such as lidocaine directly block Nav channels at the corneal nerve endings on the epithelial surface. However, concerns about the risks of delayed epithelial healing and neurotoxicity limit its use to in-office procedures [140,141,142]. Topical low-dose sodium channel blockers are therefore currently under investigation for long-term use without compromising corneal integrity.

Although still under further investigation, the use of these neuromodulatory agents represents a shift in the DED treatment paradigm. Instead of purely symptomatic and anti-inflammatory treatment, there is a movement towards targeted modulation of aberrant ion channel activity and neuronal hyperexcitability. With this shift, there is a need for careful patient selection and dosing, as over-suppression of corneal nerve function may further increase the risk of neurotrophic complications.

### 5.5. Summary and Therapeutic Implications

**Scheme 2:**
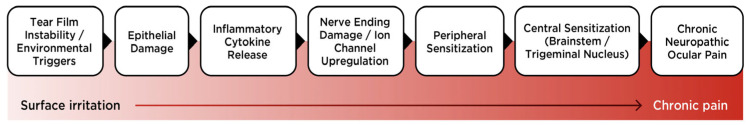
Description of the pathophysiological progression from surface-level irritation to chronic neuropathic ocular pain as seen in dry eye disease (DED).

Most current therapies do not directly modulate ion channels, but they all exert secondary effects on ion channel expression or activation, particularly in the context of nociception and inflammation [57]. Therefore, the therapeutic efficacy of the described therapies may depend on their ability to modulate the corneal microenvironment by altering the sensitivity and response of ion channels [57]. Failures in current treatment, specifically in patients who present with a neuropathic pain phenotype, may also reflect a need to better address the underlying ion channel dysregulation. While current therapies may alleviate certain aspects of ocular surface irritation, they often fail to address the underlying neuronal mechanisms that contribute to chronic ocular pain. As a result, many patients remain symptomatic despite conventional treatments that primarily target tear film deficiency or inflammation without addressing the neuropathic components of chronic ocular pain (Scheme 2). Recognizing the relationship between current therapies and their effects on ion channels can lay the groundwork for more targeted therapies already in development or in early clinical trials.

## 6. Emerging Ion Channel-Targeted Therapies

More recent research and development have been guided by the recognition that conventional treatments for dry eye diseases (DED) and neuropathic ocular pain fail to address the underlying molecular mechanisms in a significant subset of patients. These new therapeutics are more selective and directly modulate ion channels involved in sensory transduction, inflammation, and neural plasticity. Specifically, ion channels implicated in DED pathogenesis, such as TRP channels, voltage-gated sodium channels, and P2X purinergic receptors, are being investigated as promising therapeutic targets [48,57,134,139]. There have been increasing efforts to assess the efficacy of these therapies, spanning preclinical studies, early-phase clinical trials, and novel drug-delivery innovations.

### 6.1. TRPM8 Agonists

The most prominent development in ion channel modulatory therapies is the targeting of the TRPM8 channel, a cold-sensitive ion channel expressed on a subset of corneal sensory nerves [59,101,143,144]. It has also been found that TRPM8 activity can counter pro-nociceptive signals from TRPV1 and TRPA1, thus modulating pain perception [100,145,146,147]. Rather than suppressing inflammation or lubricating the eye, pharmacological stimulation of TRPM8 aims to restore impaired sensory input that is critical for tear reflexes and corneal health [101].

Alcon has recently developed a TRPM8 agonist, AR-15512, which has since been transferred to Aerie Pharmaceuticals and has shown promising potential in phase 2 clinical trials [148]. The drug is administered topically and mimics a cooling sensation, stimulating TRPM8-positive nerves and physiologically increasing tear secretion. Clinical trials have found that AR-15512 improved objective signs, such as the Schirmer score, and subjective symptoms, such as ocular discomfort in DED [148]. Two key Phase 3 trials (COMET-2 and COMET-3) enrolled more than 930 subjects that were randomized 1:1 to AR-15512 0.003% or vehicle. The primary endpoint was defined as patients achieving at least a 10 mm increase in unanesthetized Schirmer score at Day 14 and was achieved with highly significant differences between active and vehicle groups (*p* < 0.0001) [149]. In these two studies, 42.6% and 53.2% of AR-155512-treated patients achieved a ≥10 mm Schirmer increase at Day 14 versus 8.2–21.9% of vehicle-treated patients, which corresponds to absolute response differences of approximately 26.7–43.8 percentage points (*p* < 0.0001) [149]. It was also found that tear production increased as early as Day 1 and was sustained through Day 90. The most frequently reported adverse event to this treatment was mild burning or stinging at the instillation site, with no serious ocular adverse events, supporting safe chronic use [150]. Additional trials are underway to characterize long-term efficacy and safety. More recently, the FDA has approved the first TRPM8 agonist for DED treatment [148,151] under the nonproprietary name acoltremon and is marketed as TRYPTYR (acoltremon aophtalmic solution 0.003%) as the first-in-class TRPM8-targeting modulatory therapy for DED. Additionally, next generation TRPM8 agonists are entering early development, such as IVW-1001, which is a water-soluble, highly selective TRPM8 agonist formulated for optimized ocular residence time. IVW-1001 has received FDA clearance of and IND to begin Phase 1/2 clinical trials in DED [152]. Preliminary data suggests increased basal tearing and relief of neuropathic-type ocular discomfort without notable significant safety signals.

### 6.2. TRPV1 Antagonists

The transient receptor potential vanilloid 1 (TRPV1) channel is another major target in ocular surface disease [98,117,153]. TRPV1 is a polymodal nociceptor stimulated by heat, acid, capsaicin, and pro-inflammatory mediators. During inflammation, TRPV1 is upregulated in corneal nerves and epithelial cells [93]. Upon stimulation, there is a release of substance P and calcitonin gene-related peptide (CGRP), which contribute to burning pain sensation, photophobia, and neurogenic inflammation [59].

GlaxoSmithKline has developed a selective TRPV1 antagonist, SB-705498, that demonstrates analgesic properties in inflammatory and neuropathic pain models. In a randomized crossover study in 16 healthy volunteers, SB-705498 was shown to reduce capsaicin-induced flare and itch, although the mean reduction in peak itch versus placebo was not dramatic (−0.64 units on a 100 mm visual analog scale; 95% CI −3.71 to 2.44) [154], and ocular applications were limited. Similarly, Janssen has developed JNJ-38893777, also a selective TRPV1 antagonist that has been evaluated in preclinical pain models and early human studies. Both have been proposed as a potential therapeutic for ocular discomfort in DED and have shown some efficacy. However, there are larger concerns about the disruption of thermosensory function, which may compromise protective blink reflexes or contribute to corneal hypesthesia with improper dosage [155].

A recent phase 2 randomized, double-masked study of the topical TRPV1 antagonist SJP-0132 in patients with DED (SJP-0132 0.1%, 0.3%, or 1.0% versus placebo for 4 weeks) found dose-dependent efficacy. The primary endpoint was defined as a change from baseline to Week 4 in total corneal fluorescein staining (CFS) score, and the study reported an improvement in the 0.3% group versus placebo as well as statistically significant improvements versus placebo in selected secondary endpoints, which include symptom and quality-of-life measures that were evident after 1 week and maintained through Week 4 [156]. Subgroup analyses showed that SJP-0132 at 0.3% had the greatest treatment effect across baseline ocular characteristic strata, indicating 0.3% as the optimal dose for further clinical development. SJP-0132 was well tolerated across all dosages and had a safety profile comparable to placebo [156].

Among the TRPV1-targeted therapies in development, SAF312 (Libvutan/libvatrep) presents as the most clinically advanced candidate. A first-in-human study in healthy volunteers was conducted to assess the safety and tolerability of single and multiple ascending doses of topical SAF312 up to 2.5%. This dosage was administered 8 times daily for 7 days and was well tolerated, with no serious adverse events, no dose-limiting toxicity, and no clinically relevant changes in corneal esthesiometry [157]. In another phase 2 proof-of-concept trial in 40 subjects undergoing photorefractive keratectomy, administration of SAF312 at 2.5% four times daily for 3 days achieved the primary endpoints of reducing mean ocular pain visual analog scale (VAS) scores by −11.13 points versus vehicle (−25%) at 6 hours postoperatively and by −8.56 points versus vehicle (−22%) over the 0–12 h period [158]. Additionally, mean VAS pain scores were more consistently decreased with SAF312 than with vehicle from 1 to 30 h after surgery. Fewer patients requested rescue oral morphine (ORM) in the SAF312 group over 0–72 h, and there were fewer opioid users in the first 24 h. There were no reported serious ocular or systemic adverse events and no delay in epithelial healing. There was a lower incidence of grade 4 conjunctival hyperemia at 24 h in SAF312-treated eyes compared with vehicle [158]. SAF312 has now completed phase 2/3 clinical programs in DED-related and postoperative ocular surface pain, with current data demonstrating its potential to significantly reduce ocular pain and mitigate ocular inflammation while preserving corneal sensation.

### 6.3. Nav Channel Inhibitors

Voltage-gated sodium (Nav) channels are crucial for action potential initiation and conduction in nociceptive neurons [159,160]. In particular, Nav1.7, Nav1.8, and Nav1.9 channels are overexpressed or hyperactive in chronic pain states, including neuropathic pain post-refractive surgery or chronic inflammation [110,159,161].

Small-molecule Nav channel inhibitors are being developed as peripheral-selective analgesics that avoid sedative and systemic adverse effects of traditional systemic pain medications [162]. Vertex Pharmaceuticals has demonstrated that its Nav1.8-selective inhibitor, VX-150, shows target engagement and analgesic effects in human experimental pain and peripheral neuropathy studies. In a phase 1 randomized, double-blind, crossover trial in 24 healthy volunteers, a single oral dose of VX-150 1250 mg showed a significant increase in cold pressor pain tolerance time versus placebo at 2, 4, 7, and 10 h post-dose, with the largest least-squares mean difference at 4 h [163]. A treatment effect of approximately 20–30 s on cold pressor tolerance was reported across multiple time points (*p* < 0.05) and was well tolerated, with headache as the most reported event. In subsequent peripheral neuropathy programs, Nav1.8 inhibitors VX-150 and suzetrigine reported mean reductions in numeric pain rating scale scores of about −2.0 points versus −1.9 points with placebo over 12 weeks [163]. Similarly, Teva and Xenon Pharmaceuticals have also developed the topical Nav1.7/Nav1.8 inhibitor TV-45070 (funapide) and have evaluated its efficacy in patients with post-herpetic neuralgia (PHN) [164]. While the clinical development of funapide has focused on PHN and other peripheral neuropathies, its topical and peripherally acting mechanisms have also been proposed for ocular surface applications. As it currently stands, few Nav inhibitors have been directly assessed in DED cohorts, but case reports and small series suggest that topical lidocaine or systemic sodium-channel-modulating agents like carbamazepine may reduce spontaneous ocular pain in patients with documented corneal nerve hyperexcitability or neuropathic-like ocular pain features [52]. Current work with Nav inhibitors for ocular pain is focused on optimizing delivery to the corneal subbasal nerve plexus and on therapeutic dosages that suppress pathological high-frequency firing while preserving normal nociceptive input required for homeostatic blink and tear reflexes [165,166].

### 6.4. P2X3 Antagonists

The P2X3 receptor is a subtype of the ATP-gated purinergic ion channel family [167]. P2X3 and P2X2/3 receptors are enriched in small-diameter nociceptive neurons of trigeminal and dorsal root ganglia, and a subset of these P2X3-expressing trigeminal neurons innervates the ocular surface [111]. During epithelial damage or immune cell activation, ATP is released and activates P2X3 receptors, leading to spontaneous neuronal firing and increased sensitivity to mechanical or osmotic stimuli [167].

Gefapixant (MK-7264) is a selective P2X3 antagonist developed by Merck and has been successful in clinical trials for chronic cough [168]. While it has yet to be evaluated for ocular disorders, there are many shared pathophysiological features between chronic cough and dry eye that suggest possible concurrent applicability [111,167,169,170]. Both chronic cough and dry eye have hypersensitive peripheral sensory neurons, and antagonism of P2X2 may reduce aberrant nerve firing. Preclinical studies in ocular inflammation models have established that P2X3 antagonism reduces pain and tear film instability, warranting further studies for its applicability in clinical trials in DED [166].

## 7. Clinical Translation and Challenges

The development of ion channel-targeted therapies for dry eye disease (DED) and neuropathic ocular pain indicates an important shift in the therapeutic paradigm for DED, with a focus on mechanism-based treatments that can be tailored to individual pathophysiology [57,136,139,171]. While there is much potential, numerous scientific, regulatory, and practical challenges remain before full clinical translation can occur. Understanding these obstacles is essential for guiding continued research aimed at improving trial design and optimizing patient outcomes.

### 7.1. Heterogeneity of Disease Phenotypes

The primary challenge in translating ion channel-based therapies to the clinical setting is the heterogeneous nature of DED and ocular pain syndromes. As highlighted in the introduction of this review, DED has a broad spectrum of etiologies—including evaporative dry eye, aqueous tear deficiency, surgical trauma, autoimmune inflammation, and idiopathic neuropathy—with shared symptoms but varying underlying mechanisms [52,172]. For example, a patient with meibomian gland dysfunction can present with evaporative tear loss with minimal nerve pathophysiology, whereas a patient with post-LASIK dysesthesia may have significant nerve damage but with preserved tear production [171,173].

This heterogeneity both complicates diagnosis and clinical trial enrollment, as treatments that target ion channel modulation may only benefit subsets of patients with specific neurosensory phenotypes [57,136,139,171]. Patient selection becomes difficult without objective biomarkers of ion channel expression or function, which increases the risk of trial failure due to unrepresentative heterogeneity in therapeutic response. As such, current research is focused on developing diagnostic tools, such as in vivo confocal microscopy, tear proteomics, and electrophysiological assays, to accurately phenotype patients for appropriate treatments [174,175].

### 7.2. Safety and Off-Target Effects

Ion channels are often widely expressed across multiple tissues and systems, including the cornea, trigeminal ganglia, brain, skin, and cardiac tissues. Their widespread presence in the body highlights their importance in sensory physiology but also raises concerns about off-target effects [58,176].

Off-target effects of ion channel-based therapies for dry eye disease can be broadly divided into local ocular events due to topical delivery and systemic effects arising from wider tissue expression of these channels. For example, TRPM8 agonists have been reported to cause mild and transient instillation-site burning, hyperemia, and blurred with topical application due to nonspecific activation of corneal nerve terminals and epithelium rather than the intended neuromodulatory action [177]. Additionally, while topical formulations offer the advantage of targeted delivery to the ocular surface, permeability across corneal barriers, especially for larger or lipophilic molecules, remains a challenge.

Systemic concerns arise from non-ocular indications that underline important safety concerns. For example, TRPV1 antagonists have been associated with impaired thermoregulation and reduced heat pain perception that can lead to increased risk of accidental thermal injury [155]; less selective systemic sodium channel blockers could also cause central nervous system side effects or arrhythmias by affecting NaV isoforms in the brain or myocardium [178,179,180]. P2X3 antagonists are known to cause dysgeusia and ageusia in systemic use due to blockade of taste-related signaling and broader TRP modulation that may interfere with protective nociceptive and inflammatory responses in other organs.

Therefore, corneal specificity with minimal systemic absorption must be an important parameter for the continued development of ion channel therapies. Sustained-release vehicles such as hydrogels, nanoparticles, and microneedle patches may be the answer to both requirements and are being studied for improved bioavailability and localized delivery with limited systemic exposure [181,182].

### 7.3. Clinical Trial Design

Designing effective clinical trials for ion channel-based therapies also requires new trial frameworks. While standard endpoints such as the Schirmer test, corneal staining, and ocular surface disease index (OSDI) are useful in other clinical contexts, they often do not correlate well with neuropathic pain symptoms or neurosensory dysfunction [5,183,184,185,186]. For ion channel-targeting drugs, more relevant testing could include quantitative sensory testing (QST), blink reflex latency, tear meniscus dynamics, or patient-reported outcomes focused on pain descriptors [187].

Furthermore, the subjective nature of symptom reporting in ocular pain trials can result in placebo effects. Trials must be carefully designed with sufficient power and validated pain-specific scales [139,184,188]. Additionally, investigators can use enrichment strategies, such as including only patients with confirmed corneal nerve abnormalities, to increase the reliability of detecting true drug effects [175,189].

### 7.4. Patient and Physician Education

The translation of ion channel-based therapies requires a change in how patients and clinicians understand dry eye and ocular pain. Many patients describe eye discomfort as “dryness” and expect lubricative treatments to offer relief. Physicians may also be less cognizant of neuropathic pain mechanisms, particularly when clinical examination does not reveal overt signs of inflammation or epithelial disruption [134].

Integration of neurosensory evaluation into routine eye exams can not only give clinicians a more comprehensive understanding of their patients’ DED pathophysiology but can also be an opportunity to help patients understand the nerve-based components of their disease. This understanding can also help them manage their expectations about treatment timelines and outcomes [136,190]. It will also be crucial to educate clinicians on how to identify appropriate candidates for ion channel-modulating therapies, as well as on monitoring patients for potential side effects to ensure the safe and effective integration of these novel therapies [54].

## 8. Future Directions and Conclusions

As we further delineate the molecular mechanisms of dry eye disease (DED) and neuropathic ocular pain, there will be a parallel evolution of therapeutic strategies that utilize precise, pathophysiological reasoning. Ion channel-based therapies are at the forefront of this shift in the DED treatment paradigm from solely symptom suppression to targeting specific neural and inflammatory disease contributors [57,160,171].

One particularly promising direction involves the continued research and development of topical ion channel modulators, with a focus on increasing ocular bioavailability and improving targeted delivery systems. Exploration of nanoformulations, hydrogel carriers, and contact lens-based drug delivery platforms may provide a delivery method that ensures precise targeting of corneal nerves with minimized systemic absorption and consequent side effects [191]. Personalized dosing regimens and sustained-release technologies can also improve patient compliance, thereby enhancing treatment efficacy.

Another area for advancement is the development of diagnostic tools and biomarkers of neurosensory dysfunction. In vivo confocal microscopy, corneal esthesiometry, ocular surface proteomics, and functional neuroimaging could provide greater detail on nerve integrity, inflammatory status, and sensory profiles, enabling targeted treatment [175,192]. Additionally, it would allow for earlier diagnoses and disease monitoring in response to therapy.

Sex and age-based differences must also be addressed in future research. Women and older adults have disproportionately higher DED incidence and may also show differential responses to ion channel-targeted therapies due to hormonal or molecular differences in nerve signaling pathways [29,30]. Preclinical models and clinical trials should implement appropriate stratification to evaluate these differences for equitable and effective treatment across all demographics. 

Combination therapies, pairing ion channel modulators with anti-inflammatory agents, tear stabilizers, or neurotrophic factors, may also be developed to address the multifactorial nature of DED. For example, a TRPM8 agonist could be used in conjunction with cyclosporine to enhance basal tear production while simultaneously suppressing T-cell-mediated inflammation [122,193]. A synergistic approach may allow for lower dosing, reduced side effects, and more durable outcomes for patients with a mixed etiology of nociceptive and neuropathic pain.

Ion channels have the potential to be a transformative class of therapeutic targets for dry eye disease and neuropathic ocular pain. Modulation of sensory and inflammatory pathways provides not only symptomatic relief but also molecular restoration of ocular surface homeostasis and nerve function. In addition to the scientific advancement of these therapies, successful clinical translation requires the establishment of collaborative networks between clinicians, neuroscientists, and pharmaceutical developers. Not only is there a need for interdisciplinary research to achieve efficient drug development pipelines, refined clinical trial endpoints, and facilitation of knowledge transfer from bench to bedside, but there is also a need for a thorough shared understanding of these new therapies for effective education of providers and patients.

## Data Availability

No new data were created or analyzed in this study. Data sharing is not applicable to this article.

## Abbreviations

The following abbreviations are used in this manuscript:

DED	Dry eye disease
EDE	Evaporative dry eye
ADDE	Aqueous deficient dry eye
TRP	Transient receptor potential
TFOS	Tear Film and Ocular Surface Society
DEWS	Dry Eye Workshop
LASIK	Laster-assisted in situ keratomileusis
PRK	Photorefractive keratectomy
MGD	Meibomian gland dysfunction
TRPM8	Transient receptor potential melastatin 8
TRPA1	Transient receptor potential ankyrin 1
TRPV1	Transient receptor potential vanilloid 1
TRPV4	Transient receptor potential cation channel subfamily V member 4
MAPK	Mitogen-activated protein kinase
NF-ΚB	Nuclear factor ΚB
EGFR	Epidermal growth factor receptor
TCA	Tricyclic antidepressants
SNRI	Serotonin–norepinephrine reuptake inhibitors
VGCC	Voltage-gated calcium channels
OSDI	Ocular surface disease index
QST	Quantitative sensory testing

## References

[1] Craig J.P., Nichols K.K., Akpek E.K., Caffery B., Dua H.S., Joo C.-K., Liu Z., Nelson J.D., Nichols J.J., Tsubota K. (2017). TFOS DEWS II Definition and Classification Report. Ocul. Surf..

[2] Craig J.P., Nelson J.D., Azar D.T., Belmonte C., Bron A.J., Chauhan S.K., De Paiva C.S., Gomes J.A.P., Hammitt K.M., Jones L. (2017). TFOS DEWS II Report Executive Summary. Ocul. Surf..

[3] Deo N., Nagrale P. (2024). Dry Eye Disease: An Overview of Its Risk Factors, Diagnosis, and Prevalence by Age, Sex, and Race. Cureus.

[4] Aggarwal S., Galor A. (2018). What’s New in Dry Eye Disease Diagnosis? Current Advances and Challenges. F1000Research.

[5] Bartlett J.D., Keith M.S., Sudharshan L., Snedecor S.J. (2015). Associations between Signs and Symptoms of Dry Eye Disease: A Systematic Review. Clin. Ophthalmol..

[6] Uchino M., Schaumberg D.A. (2013). Dry Eye Disease: Impact on Quality of Life and Vision. Curr. Ophthalmol. Rep..

[7] Martinescu G., Bogdanici C.M., Pavel I.A., Ciocoiu M. (2022). Difficulties in Performing Daily Activities in Patients with Dry Eye before and after Treatment. Medicina.

[8] Deschamps N., Ricaud X., Rabut G., Labbé A., Baudouin C., Denoyer A. (2013). The Impact of Dry Eye Disease on Visual Performance While Driving. Am. J. Ophthalmol..

[9] Narendra K., Singh S.K., Deepa C.K., Meghana S., Akanth K.R., Manjushree M., Raajasubramaniyan D., Srinivasan S., Murali R., Sowbhagya H.N. (2025). Exploring New Frontiers in Dry Eye Disease: Treatments, Mechanisms, and Diagnostic Innovations a Comprehensive Review. Asp. Mol. Med..

[10] Golden M.I., Meyer J.J., Zeppieri M., Patel B.C. (2025). Dry Eye Syndrome. StatPearls.

[11] Cho J., Bell N., Botzet G., Vora P., Fowler B.J., Donahue R., Bush H., Taylor B.K., Albuquerque R.J.C. (2019). Latent Sensitization in a Mouse Model of Ocular Neuropathic Pain. Transl. Vis. Sci. Technol..

[12] Asiedu K. (2022). Role of Ocular Surface Neurobiology in Neuronal-Mediated Inflammation in Dry Eye Disease. Neuropeptides.

[13] Ebrahimiadib N., Yousefshahi F., Abdi P., Ghahari M., Modjtahedi B.S. (2020). Ocular Neuropathic Pain: An Overview Focusing on Ocular Surface Pains. Clin. Ophthalmol..

[14] Liu H., Begley C., Chen M., Bradley A., Bonanno J., McNamara N.A., Nelson J.D., Simpson T. (2009). A Link between Tear Instability and Hyperosmolarity in Dry Eye. Investig. Ophthalmol. Vis. Sci..

[15] Harrell C.R., Feulner L., Djonov V., Pavlovic D., Volarevic V. (2023). The Molecular Mechanisms Responsible for Tear Hyperosmolarity-Induced Pathological Changes in the Eyes of Dry Eye Disease Patients. Cells.

[16] Kaur K., Stokkermans T.J. (2025). Meibomian Gland Disease. StatPearls.

[17] Rolando M., Merayo-Lloves J. (2022). Management Strategies for Evaporative Dry Eye Disease and Future Perspective. Curr. Eye Res..

[18] Al-Mohtaseb Z., Schachter S., Shen Lee B., Garlich J., Trattler W. (2021). The Relationship Between Dry Eye Disease and Digital Screen Use. Clin. Ophthalmol..

[19] Venugopal A., Soni A., Ravindran M., Siddiq M.U. (2025). Screen Strain: The Prevalence of Digital Eye Strain and Dry Eye Symptoms in Ophthalmologists Using Video Display Terminals. TNOA J. Ophthalmic Sci. Res..

[20] Yahalomi T., Achiron A., Arnon R., Stanescu N., Pikkel J. (2023). Dry Eye Disease Following LASIK, PRK, and LASEK: An Observational Cross-Sectional Study. J. Clin. Med..

[21] Calvillo M.P., McLaren J.W., Hodge D.O., Bourne W.M. (2004). Corneal Reinnervation after LASIK: Prospective 3-Year Longitudinal Study. Investig. Ophthalmol. Vis. Sci..

[22] Konomi K., Chen L.-L., Tarko R.S., Scally A., Schaumberg D.A., Azar D., Dartt D.A. (2008). Preoperative Characteristics and a Potential Mechanism of Chronic Dry Eye after LASIK. Investig. Ophthalmol. Vis. Sci..

[23] Bower K.S., Sia R.K., Ryan D.S., Mines M.J., Dartt D.A. (2015). Chronic Dry Eye in Photorefractive Keratectomy and Laser in Situ Keratomileusis: Manifestations, Incidence, and Predictive Factors. J. Cataract. Refract. Surg..

[24] Naderi K., Gormley J., O’Brart D. (2020). Cataract Surgery and Dry Eye Disease: A Review. Eur. J. Ophthalmol..

[25] Peterson D.C., Hamel R.N. (2025). Corneal Reflex. StatPearls.

[26] Shaheen B., Bakir M., Jain S. (2014). Corneal Nerves in Health and Disease. Surv. Ophthalmol..

[27] Mikalauskiene L., Grzybowski A., Zemaitiene R. (2021). Ocular Surface Changes Associated with Ophthalmic Surgery. J. Clin. Med..

[28] Gorimanipalli B., Khamar P., Sethu S., Shetty R. (2023). Hormones and Dry Eye Disease. Indian J. Ophthalmol..

[29] Nuzzi R., Caselgrandi P. (2022). Sex Hormones and Their Effects on Ocular Disorders and Pathophysiology: Current Aspects and Our Experience. Int. J. Mol. Sci..

[30] Sullivan D.A. (2004). Tearful Relationships? Sex, Hormones, the Lacrimal Gland, and Aqueous-Deficient Dry Eye. Ocul. Surf..

[31] Hat K., Planinić A., Ježek D., Kaštelan S. (2023). Expression of Androgen and Estrogen Receptors in the Human Lacrimal Gland. Int. J. Mol. Sci..

[32] He B., Iovieno A., Etminan M., Kezouh A., Yeung S.N. (2022). Effects of Hormonal Contraceptives on Dry Eye Disease: A Population-Based Study. Eye.

[33] Peck T., Olsakovsky L., Aggarwal S. (2017). Dry Eye Syndrome in Menopause and Perimenopausal Age Group. J. Midlife Health.

[34] Yu K., Bunya V., Maguire M., Asbell P., Ying G.-S. (2021). Dry Eye Assessment and Management Study Research Group Systemic Conditions Associated with Severity of Dry Eye Signs and Symptoms in the Dry Eye Assessment and Management Study. Ophthalmology.

[35] Roszkowska A.M., Oliverio G.W., Aragona E., Inferrera L., Severo A.A., Alessandrello F., Spinella R., Postorino E.I., Aragona P. (2021). Ophthalmologic Manifestations of Primary Sjögren’s Syndrome. Genes.

[36] Patel S., Mittal R., Kumar N., Galor A. (2023). The Environment and Dry Eye—Manifestations, Mechanisms, and More. Front. Toxicol..

[37] Muñoz-Villegas P., García-Sánchez G., Jauregui-Franco R.O., Quirarte-Justo S., Sánchez-Ríos A., Olvera-Montaño O. (2024). Influence of Environmental Factors with Clinical Signs and Symptoms in the Management of Dry Eye Disease. Clin. Ophthalmol..

[38] Ghach W., Bakkar M.M., Aridi M., Alebrahim M.A. (2025). Symptomatic Dry Eye Disease (DED) in Cohort of Contact Lens Wearers in Jordan. PLoS ONE.

[39] García-Marqués J.V., Talens-Estarelles C., García-Lázaro S., Cerviño A. (2022). The Effects of Soft Contact Lens Wear on the Tear Film and Meibomian Gland Drop-Out and Visibility. Life.

[40] Guo M., Diaz G., Yu Y., Patel C., Farrar J., Asbell P., Ying G.-S. (2024). Association between Systemic Medication Use and Severity of Dry Eye Signs and Symptoms in the DRy Eye Assessment and Management (DREAM) Study. Ocul. Surf..

[41] Datta S., Baudouin C., Brignole-Baudouin F., Denoyer A., Cortopassi G.A. (2017). The Eye Drop Preservative Benzalkonium Chloride Potently Induces Mitochondrial Dysfunction and Preferentially Affects LHON Mutant Cells. Investig. Ophthalmol. Vis. Sci..

[42] Goldstein M.H., Silva F.Q., Blender N., Tran T., Vantipalli S. (2022). Ocular Benzalkonium Chloride Exposure: Problems and Solutions. Eye.

[43] Ivakhnitskaia E., Souboch V., Dallacasagrande V., Mizerska K., Souboch E., Sarkar J., Guaiquil V.H., Tseng K.Y., Hirata H., Rosenblatt M.I. (2022). Benzalkonium Chloride, a Common Ophthalmic Preservative, Compromises Rat Corneal Cold Sensitive Nerve Activity. Ocul. Surf..

[44] Wong J., Lan W., Ong L.M., Tong L. (2011). Non-Hormonal Systemic Medications and Dry Eye. Ocul. Surf..

[45] Leonardi A., Bozkurt B., Silva D., Mortz C.G., Baudouin C., Atanaskovic-Markovic M., Sharma V., Doan S., Agarwal S., Pérez-Formigo D. (2025). Drug-Induced Periocular and Ocular Surface Disorders: An EAACI Position Paper. Allergy.

[46] Hossain P. (2024). Reducing the Stress of Corneal Neuropathic Pain: ‘Pain without Stain’. Eye.

[47] Rosenthal P., Baran I., Jacobs D.S. (2009). Corneal Pain without Stain: Is It Real?. Ocul. Surf..

[48] Moshirfar M., Benstead E.E., Sorrentino P.M., Tripathy K. (2025). Ocular Neuropathic Pain. StatPearls.

[49] Fakih D., Zhao Z., Nicolle P., Reboussin E., Joubert F., Luzu J., Labbé A., Rostène W., Baudouin C., Mélik Parsadaniantz S. (2019). Chronic Dry Eye Induced Corneal Hypersensitivity, Neuroinflammatory Responses, and Synaptic Plasticity in the Mouse Trigeminal Brainstem. J. Neuroinflammation.

[50] Puja G., Sonkodi B., Bardoni R. (2021). Mechanisms of Peripheral and Central Pain Sensitization: Focus on Ocular Pain. Front. Pharmacol..

[51] Andersen H.H., Yosipovitch G., Galor A. (2017). Neuropathic Symptoms of the Ocular Surface: Dryness, Pain, and Itch. Curr. Opin. Allergy Clin. Immunol..

[52] Galor A., Moein H.-R., Lee C., Rodriguez A., Felix E.R., Sarantopoulos K.D., Levitt R.C. (2018). Neuropathic Pain and Dry Eye. Ocul. Surf..

[53] McCann P., Kruoch Z., Lopez S., Malli S., Qureshi R., Li T. (2024). Interventions for Dry Eye An Overview of Systematic Reviews. JAMA Ophthalmol..

[54] Betz J., Galor A. (2025). Navigating the Dry Eye Therapeutic Puzzle: A Mechanism-Based Overview of Current Treatments. Pharmaceuticals.

[55] Tong L., Liu Z., Şahin A., Gümüş K., Messmer E.M., Benítez-del-Castillo J.M., Labetoulle M., Chan C.C., Periman L.M. (2025). Topical Pharmacologic Treatments for Dry Eye Disease: A Systematic Review. Ocul. Surf..

[56] Costigan M., Scholz J., Woolf C.J. (2009). Neuropathic Pain. Annu. Rev. Neurosci..

[57] Harrell C.R., Volarevic V. (2024). Ion Channels as Potential Drug Targets in Dry Eye Disease and Their Clinical Relevance: A Review. Cells.

[58] Ashok N., Khamar P., D’Souza S., Gijs M., Ghosh A., Sethu S., Shetty R. (2023). Ion Channels in Dry Eye Disease. Indian J. Ophthalmol..

[59] Schecterson L.C., Pazevic A.A., Yang R., Matulef K., Gordon S.E. (2020). TRPV1, TRPA1, and TRPM8 Are Expressed in Axon Terminals in the Cornea: TRPV1 Axons Contain CGRP and Secretogranin II; TRPA1 Axons Contain Secretogranin 3. Mol. Vis..

[60] Goldstein R.H., Barkai O., Íñigo-Portugués A., Katz B., Lev S., Binshtok A.M. (2019). Location and Plasticity of the Sodium Spike Initiation Zone in Nociceptive Terminals In Vivo. Neuron.

[61] Guzman-Aranguez A., Gasull X., Diebold Y., Pintor J. (2014). Purinergic Receptors in Ocular Inflammation. Mediat. Inflamm..

[62] Dartt D.A. (2009). Neural Regulation of Lacrimal Gland Secretory Processes: Relevance in Dry Eye Diseases. Prog. Retin. Eye Res..

[63] Wang L., Dai W., Lu L. (2011). Hyperosmotic Stress-Induced Corneal Epithelial Cell Death through Activation of Polo-like Kinase 3 and c-Jun. Investig. Ophthalmol. Vis. Sci..

[64] Garcia-Queiruga J., Pena-Verdeal H., Sabucedo-Villamarin B., Garcia-Resua C., Giraldez M.J., Yebra-Pimentel E. (2024). Temporal Progression of Entry Factors into the Vicious Circle of Dry Eye in Untreated Sufferers. Life.

[65] Baudouin C., Aragona P., Messmer E.M., Tomlinson A., Calonge M., Boboridis K.G., Akova Y.A., Geerling G., Labetoulle M., Rolando M. (2013). Role of Hyperosmolarity in the Pathogenesis and Management of Dry Eye Disease: Proceedings of the *OCEAN* Group Meeting. Ocul. Surf..

[66] Chhadva P., Goldhardt R., Galor A. (2017). Meibomian Gland Disease: The Role of Gland Dysfunction in Dry Eye Disease. Ophthalmology.

[67] Schirra F., Richards S.M., Liu M., Suzuki T., Yamagami H., Sullivan D.A. (2006). Androgen Regulation of Lipogenic Pathways in the Mouse Meibomian Gland. Exp. Eye Res..

[68] Matossian C., McDonald M., Donaldson K.E., Nichols K.K., MacIver S., Gupta P.K. (2019). Dry Eye Disease: Consideration for Women’s Health. J. Women’s Health.

[69] Farrand K.F., Fridman M., Stillman I.Ö., Schaumberg D.A. (2017). Prevalence of Diagnosed Dry Eye Disease in the United States Among Adults Aged 18 Years and Older. Am. J. Ophthalmol..

[70] Módulo C.M., Jorge A.G., Dias A.C., Braz A.M., Bertazolli-Filho R., Jordão A.A., Sérgio Marchini J., Rocha E.M. (2009). Influence of Insulin Treatment on the Lacrimal Gland and Ocular Surface of Diabetic Rats. Endocrine.

[71] de Paiva C.S., St. Leger A.J., Caspi R.R. (2022). Mucosal Immunology of the Ocular Surface. Mucosal Immunol..

[72] Stern M.E., Schaumburg C.S., Pflugfelder S.C. (2013). Dry Eye as a Mucosal Autoimmune Disease. Int. Rev. Immunol..

[73] Wu K.Y., Kulbay M., Tanasescu C., Jiao B., Nguyen B.H., Tran S.D. (2023). An Overview of the Dry Eye Disease in Sjögren’s Syndrome Using Our Current Molecular Understanding. Int. J. Mol. Sci..

[74] Gomes J.A.P., Azar D.T., Baudouin C., Efron N., Hirayama M., Horwath-Winter J., Kim T., Mehta J.S., Messmer E.M., Pepose J.S. (2017). TFOS DEWS II Iatrogenic Report. Ocul. Surf..

[75] Shtein R.M. (2011). Post-LASIK Dry Eye. Expert Rev. Ophthalmol..

[76] Zhao L., Zhang Y., Duan H., Yang T., Zhou Y., Ma B., Chen Y., Qi H. (2023). Clinical Characteristics and Tear Film Biomarkers in Patients with Chronic Dry Eye Disease After Femtosecond Laser-Assisted Laser in Situ Keratomileusis. J. Refract. Surg..

[77] Ţuru L., Alexandrescu C., Stana D., Tudosescu R. (2012). Dry Eye Disease after LASIK. J. Med. Life.

[78] Tamimi A., Sheikhzadeh F., Ezabadi S.G., Islampanah M., Parhiz P., Fathabadi A., Poudineh M., Khanjani Z., Pourmontaseri H., Orandi S. (2023). Post-LASIK Dry Eye Disease: A Comprehensive Review of Management and Current Treatment Options. Front. Med..

[79] Alamri A., Alshehri A.A.M., Alshamrani A.S.R., Alshehri A.S.A., Ali K.M.M., Alshumrani S.G.A., Rafi A.M., Ogran M.A., AlShahrani A.F.S., Mbbs S.A.A. (2024). Dry Eye After LASIK Surgery: Comprehensive Review and Update of Literature. Bahrain Med. Bull..

[80] Wang M.T.M., Power B., Xue A.L., Craig J.P. (2025). Blink Completeness and Rate in Dry Eye Disease: An Investigator-Masked, Prospective Registry-Based, Cross-Sectional, Prognostic Study. Contact Lens Anterior Eye.

[81] Britten-Jones A.C., Wang M.T.M., Samuels I., Jennings C., Stapleton F., Craig J.P. (2024). Epidemiology and Risk Factors of Dry Eye Disease: Considerations for Clinical Management. Medicina.

[82] Guerrero-Moreno A., Baudouin C., Melik Parsadaniantz S., Réaux-Le Goazigo A. (2020). Morphological and Functional Changes of Corneal Nerves and Their Contribution to Peripheral and Central Sensory Abnormalities. Front. Cell. Neurosci..

[83] Park R., Spritz S., Zeng A.Y., Erukulla R., Zavala D., Merchant T., Gascon A., Jung R., Bigit B., Azar D.T. (2025). Corneal Sensory Receptors and Pharmacological Therapies to Modulate Ocular Pain. Int. J. Mol. Sci..

[84] Yang T.-J., Yu Y., Yang J.-Y., Li J.-J., Zhu J.-Y., Vieira J.A.C., Jiang Q. (2022). Involvement of Transient Receptor Potential Channels in Ocular Diseases: A Narrative Review. Ann. Transl. Med..

[85] Koivisto A.-P., Belvisi M.G., Gaudet R., Szallasi A. (2022). Advances in TRP Channel Drug Discovery: From Target Validation to Clinical Studies. Nat. Rev. Drug Discov..

[86] Yu F.H., Catterall W.A. (2003). Overview of the Voltage-Gated Sodium Channel Family. Genome Biol..

[87] Rogers M., Tang L., Madge D.J., Stevens E.B. (2006). The Role of Sodium Channels in Neuropathic Pain. Semin. Cell Dev. Biol..

[88] Laedermann C.J., Abriel H., Decosterd I. (2015). Post-Translational Modifications of Voltage-Gated Sodium Channels in Chronic Pain Syndromes. Front. Pharmacol..

[89] Wang J.-N., Fan H., Song J.-T. (2023). Targeting Purinergic Receptors to Attenuate Inflammation of Dry Eye. Purinergic Signal..

[90] Hodges R.R., Vrouvlianis J., Scott R., Dartt D.A. (2011). Identification of P2X3 and P2X7 Purinergic Receptors Activated by ATP in Rat Lacrimal Gland. Investig. Ophthalmol. Vis. Sci..

[91] Burnstock G. (2016). P2X Ion Channel Receptors and Inflammation. Purinergic Signal..

[92] Yang S., Wu Y., Wang C., Jin X. (2022). Ocular Surface Ion-Channels Are Closely Related to Dry Eye: Key Research Focus on Innovative Drugs for Dry Eye. Front. Med..

[93] Pan Z., Wang Z., Yang H., Zhang F., Reinach P.S. (2011). TRPV1 Activation Is Required for Hypertonicity-Stimulated Inflammatory Cytokine Release in Human Corneal Epithelial Cells. Investig. Ophthalmol. Vis. Sci..

[94] Zhang F., Yang H., Wang Z., Mergler S., Liu H., Kawakita T., Tachado S.D., Pan Z., Capó-Aponte J.E., Pleyer U. (2007). Transient Receptor Potential Vanilloid 1 Activation Induces Inflammatory Cytokine Release in Corneal Epithelium through MAPK Signaling. J. Cell. Physiol..

[95] Chung M.-K., Güler A.D., Caterina M.J. (2008). TRPV1 Shows Dynamic Ionic Selectivity during Agonist Stimulation. Nat. Neurosci..

[96] Senning E.N., Gordon S.E. (2015). Activity and Ca2+ Regulate the Mobility of TRPV1 Channels in the Plasma Membrane of Sensory Neurons. eLife.

[97] Zhang F., Jara-Oseguera A., Chang T.-H., Bae C., Hanson S.M., Swartz K.J. (2018). Heat Activation Is Intrinsic to the Pore Domain of TRPV1. Proc. Natl. Acad. Sci. USA.

[98] Gou Q., Song Z., Gong Y., Li J. (2024). TRPV1 in Dry Eye Disease. Front. Biosci..

[99] Wang Z., Yang Y., Yang H., Capó-Aponte J.E., Tachado S.D., Wolosin J.M., Reinach P.S. (2011). NF-κB Feedback Control of JNK1 Activation Modulates TRPV1-Induced Increases in IL-6 and IL-8 Release by Human Corneal Epithelial Cells. Mol. Vis..

[100] Dhaka A., Murray A.N., Mathur J., Earley T.J., Petrus M.J., Patapoutian A. (2007). TRPM8 Is Required for Cold Sensation in Mice. Neuron.

[101] Corcoran P., Hollander D.A., Ousler G.W., Angjeli E., Rimmer D., Lane K., Abelson M.B. (2017). Dynamic Sensitivity of Corneal TRPM8 Receptors to Menthol Instillation in Dry Eye Versus Normal Subjects. J. Ocul. Pharmacol. Ther..

[102] Bautista D.M., Siemens J., Glazer J.M., Tsuruda P.R., Basbaum A.I., Stucky C.L., Jordt S.-E., Julius D. (2007). The Menthol Receptor TRPM8 Is the Principal Detector of Environmental Cold. Nature.

[103] Colburn R.W., Lubin M.L., Stone D.J., Wang Y., Lawrence D., D’Andrea M.R., Brandt M.R., Liu Y., Flores C.M., Qin N. (2007). Attenuated Cold Sensitivity in TRPM8 Null Mice. Neuron.

[104] Hatta A., Kurose M., Sullivan C., Okamoto K., Fujii N., Yamamura K., Meng I.D. (2019). Dry Eye Sensitizes Cool Cells to Capsaicin-Induced Changes in Activity via TRPV1. J. Neurophysiol..

[105] Migeon T., Cordovilla A., Potey A., Delarasse C., Hourcade T., Reboussin E., Olmière C., Baudouin C., Réaux-Le Goazigo A., Mélik Parsadaniantz S. (2025). TRPA1 Inhibition Reduces Ocular Pain and Corneal Neurogenic Inflammation in a Mouse Model of Dry Eye Disease. Biomed. Pharmacother..

[106] Bautista D.M., Pellegrino M., Tsunozaki M. (2013). TRPA1: A Gatekeeper for Inflammation. Annu. Rev. Physiol..

[107] Fila M., Przyslo L., Derwich M., Sobczuk P., Pawlowska E., Blasiak J. (2024). The TRPA1 Ion Channel Mediates Oxidative Stress-Related Migraine Pathogenesis. Molecules.

[108] Katagiri A., Thompson R., Rahman M., Okamoto K., Bereiter D.A. (2015). Evidence for TRPA1 Involvement in Central Neural Mechanisms in a Rat Model of Dry Eye. Neuroscience.

[109] Bennett D.L., Clark A.J., Huang J., Waxman S.G., Dib-Hajj S.D. (2019). The Role of Voltage-Gated Sodium Channels in Pain Signaling. Physiol. Rev..

[110] Delwig A., Liu G., Meng I.D., Pajouhesh H., Monteleone D., Zhou S., Mulcahy J. (2020). NaV1.7 Inhibitors Relieve Ocular Discomfort in a Rat Model of Dry Eye Disease. Investig. Ophthalmol. Vis. Sci..

[111] Staikopoulos V., Sessle B.J., Furness J.B., Jennings E.A. (2007). Localization of P2X2 and P2X3 Receptors in Rat Trigeminal Ganglion Neurons. Neuroscience.

[112] Krajewski J.L. (2020). P2X3-Containing Receptors as Targets for the Treatment of Chronic Pain. Neurotherapeutics.

[113] Yu L., Li N., Liu C., Ma B. (2011). Estrogen Altered Facial Mechanical Pain Threshold and Trigeminal P2X3 Receptor Expression. Neuro Endocrinol. Lett..

[114] Semp D.A., Beeson D., Sheppard A.L., Dutta D., Wolffsohn J.S. (2023). Artificial Tears: A Systematic Review. Clin. Optom..

[115] Labetoulle M., Benitez-del-Castillo J.M., Barabino S., Herrero Vanrell R., Daull P., Garrigue J.-S., Rolando M. (2022). Artificial Tears: Biological Role of Their Ingredients in the Management of Dry Eye Disease. Int. J. Mol. Sci..

[116] Kim M., Lee Y., Mehra D., Sabater A.L., Galor A. (2021). Dry Eye: Why Artificial Tears Are Not Always the Answer. BMJ Open Ophth..

[117] Fakih D., Migeon T., Moreau N., Baudouin C., Réaux-Le Goazigo A., Mélik Parsadaniantz S. (2022). Transient Receptor Potential Channels: Important Players in Ocular Pain and Dry Eye Disease. Pharmaceutics.

[118] Pizzano M., Vereertbrugghen A., Cernutto A., Sabbione F., Keitelman I.A., Shiromizu C.M., Aguilar D.V., Fuentes F., Giordano M.N., Trevani A.S. (2023). Ocular TRPV1 Deficiency Protects from Dry Eye-Induced Corneal Nerve Damage. bioRxiv.

[119] Hernandez E., Taisne C., Lussignol M., Esclatine A., Labetoulle M. (2021). Commercially Available Eye Drops Containing Trehalose Protect Against Dry Conditions via Autophagy Induction. J. Ocul. Pharmacol. Ther..

[120] Gallar J., Pflugfelder S., Galor A., Gupta P.K., Hamrah P. (2025). Corneal Sensory Nerve Regulation of Tear Production through Stimulation of Transient Receptor Potential Melastatin 8 (TRPM8) Channel: A Potential New Approach for Treating Dry Eye Disease. Ocul. Surf..

[121] Garrigue J.-S., Amrane M., Faure M.-O., Holopainen J.M., Tong L. (2017). Relevance of Lipid-Based Products in the Management of Dry Eye Disease. J. Ocul. Pharmacol. Ther..

[122] Bian X., Ma J., Liu Y., Feng Y., Liu Z., Zhang B., Huang B. (2025). Cyclosporine a in the Treatment of Dry Eye Disease: A Narrative Review. Front. Ophthalmol..

[123] Periman L.M., Mah F.S., Karpecki P.M. (2020). A Review of the Mechanism of Action of Cyclosporine A: The Role of Cyclosporine A in Dry Eye Disease and Recent Formulation Developments. Clin. Ophthalmol..

[124] Paton D.M. (2016). Lifitegrast: First LFA-1/ICAM-1 Antagonist for Treatment of Dry Eye Disease. Drugs Today.

[125] Perez V.L., Pflugfelder S.C., Zhang S., Shojaei A., Haque R. (2016). Lifitegrast, a Novel Integrin Antagonist for Treatment of Dry Eye Disease. Ocul. Surf..

[126] Méndez-Reséndiz K.A., Enciso-Pablo Ó., González-Ramírez R., Juárez-Contreras R., Rosenbaum T., Morales-Lázaro S.L. (2020). Steroids and TRP Channels: A Close Relationship. Int. J. Mol. Sci..

[127] Seol S.-H., Chung G. (2022). Estrogen-Dependent Regulation of Transient Receptor Potential Vanilloid 1 (TRPV1) and P2X Purinoceptor 3 (P2X3): Implication in Burning Mouth Syndrome. J. Dent. Sci..

[128] Yao K., Dou B., Zhang Y., Chen Z., Li Y., Fan Z., Ma Y., Du S., Wang J., Xu Z. (2023). Inflammation—The Role of TRPA1 Channel. Front. Physiol..

[129] Ramírez-Barrantes R., Marchant I., Olivero P. (2016). TRPV1 May Increase the Effectiveness of Estrogen Therapy on Neuroprotection and Neuroregeneration. Neural Regen. Res..

[130] Ortíz-Rentería M., Juárez-Contreras R., González-Ramírez R., Islas L.D., Sierra-Ramírez F., Llorente I., Simon S.A., Hiriart M., Rosenbaum T., Morales-Lázaro S.L. (2018). TRPV1 Channels and the Progesterone Receptor Sig-1R Interact to Regulate Pain. Proc. Natl. Acad. Sci. USA.

[131] Ervin A., Law A., Pucker A.D. (2017). Punctal Occlusion for Dry Eye Syndrome. Cochrane Database Syst. Rev..

[132] Chen K.-Y., Chan H.-C., Chan C.-M. (2025). How Effective and Safe Are Punctal Plugs in Treating Dry Eye Disease? A Systematic Review and Meta-Analysis. Contact Lens Anterior Eye.

[133] Brissette A.R., Mednick Z.D., Schweitzer K.D., Bona M.D., Baxter S.A. (2015). Punctal Plug Retention Rates for the Treatment of Moderate to Severe Dry Eye: A Randomized, Double-Masked, Controlled Clinical Trial. Am. J. Ophthalmol..

[134] Galor A., Levitt R.C., Felix E.R., Martin E.R., Sarantopoulos C.D. (2015). Neuropathic Ocular Pain: An Important yet Underevaluated Feature of Dry Eye. Eye.

[135] Rao S.K., Mohan R., Gokhale N., Matalia H., Mehta P. (2022). Inflammation and Dry Eye Disease—Where Are We?. Int. J. Ophthalmol..

[136] Watson S.L., Le D.T.-M. (2024). Corneal Neuropathic Pain: A Review to Inform Clinical Practice. Eye.

[137] Gagliano C., Avitabile A., Rusciano D. (2025). Gabapentin for Ocular Surface Disorders: Bridging Molecular Mechanisms to Therapeutic Innovation. Arch. Clin. Ophthalmol..

[138] Michael R., Jeffers J.V., Messenger W., Aref A.A. (2020). Gabapentin for Presumed Neuropathic Ocular Pain. Am. J. Ophthalmol. Case Rep..

[139] Patel S., Mittal R., Sarantopoulos K.D., Galor A. (2022). Neuropathic Ocular Surface Pain: Emerging Drug Targets and Therapeutic Implications. Expert Opin. Ther. Targets.

[140] Leslie L., Liu S.-H., Kuo I.C. (2025). Topical Ophthalmic Anesthetics for Corneal Abrasions: Findings from a Cochrane Systematic Review and Meta-Analysis. Commun. Med..

[141] Shipman S.B., Painter K.A. (2021). A Review of the Effectiveness and Safety of Topical Anesthetics in Corneal Abrasions. J. Anesthesiol. Pain Ther..

[142] Miller D.D., Wagner I.V., Ten Hulzen R.D., Dorairaj S., Mashayekhi A., Krambeer C., Boopathiraj N., Stewart M. (2024). Delayed Corneal Healing After the Use of Topical Ophthalmic Anesthetics. Cureus.

[143] Piña R., Ugarte G., Campos M., Íñigo-Portugués A., Olivares E., Orio P., Belmonte C., Bacigalupo J., Madrid R. (2019). Role of TRPM8 Channels in Altered Cold Sensitivity of Corneal Primary Sensory Neurons Induced by Axonal Damage. J. Neurosci..

[144] Li F., Yang W., Jiang H., Guo C., Huang A.J.W., Hu H., Liu Q. (2019). TRPV1 Activity and Substance P Release Are Required for Corneal Cold Nociception. Nat. Commun..

[145] Kaido M., Inoue S., Kawashima M., Ishida R., Nakamura S., Tsubota K. (2021). Role of Transient Receptor Potential Melastatin 8 Activity in Menthol-Induced Cold Sensitivity and Its Qualitative Perception in Dry Eye. Ocul. Surf..

[146] Hirata H., Oshinsky M.L. (2011). Ocular Dryness Excites Two Classes of Corneal Afferent Neurons Implicated in Basal Tearing in Rats: Involvement of Transient Receptor Potential Channels. J. Neurophysiol..

[147] Quallo T., Vastani N., Horridge E., Gentry C., Parra A., Moss S., Viana F., Belmonte C., Andersson D.A., Bevan S. (2015). TRPM8 Is a Neuronal Osmosensor That Regulates Eye Blinking in Mice. Nat. Commun..

[148] Maharjan E.K., Reports O.S. Promising Results from Phase 2b Study of AR-15512 for the Treatment of Dry Eye|Ophthalmology Times—Clinical Insights for Eye Specialists. https://www.ophthalmologytimes.com/view/promising-results-from-phase-2b-study-of-ar-15512-for-the-treatment-of-dry-eye.

[149] Alcon Announces Positive Topline Results from Phase 3 COMET Trials of AR-15512, a Novel Topical Drug Candidate for Dry Eye|Alcon. https://www.alcon.com/media-release/alcon-announces-positive-topline-results-phase-3-comet-trials-ar-15512-novel-topical/.

[150] Iapoce C. AR-15512 for Dry Eye Disease Achieves Primary Endpoint in Phase 3 COMET Trials|HCPLive. https://www.hcplive.com/view/ar-15512-achieves-primary-endpoint-phase-3-comet-trials-dry-eye-disease.

[151] Mullard A. (2025). FDA Approves First-in-Class TRPM8 Ion Channel Agonist for Dry Eye Disease. Nat. Rev. Drug Discov..

[152] Liang B., Short B.G., Wang W., Zhu J., Pan Y., Fon J., Hong L., Yao Z. (2024). Nonclinical Development of a Novel Therapeutic Ophthalmic Eyelid Wipe to Treat Dry Eye Disease. Investig. Ophthalmol. Vis. Sci..

[153] Rahman M.M., Jo Y.-Y., Kim Y.H., Park C.-K. (2024). Current Insights and Therapeutic Strategies for Targeting TRPV1 in Neuropathic Pain Management. Life Sci..

[154] Gibson R.A., Robertson J., Mistry H., McCallum S., Fernando D., Wyres M., Yosipovitch G. (2014). A Randomised Trial Evaluating the Effects of the TRPV1 Antagonist SB705498 on Pruritus Induced by Histamine, and Cowhage Challenge in Healthy Volunteers. PLoS ONE.

[155] Garami A., Pakai E., McDonald H.A., Reilly R.M., Gomtsyan A., Corrigan J.J., Pinter E., Zhu D.X.D., Lehto S.G., Gavva N.R. (2018). TRPV1 Antagonists That Cause Hypothermia, Instead of Hyperthermia, in Rodents: Compounds’ Pharmacological Profiles, in Vivo Targets, Thermoeffectors Recruited and Implications for Drug Development. Acta Physiol..

[156] Hori Y., Wada T., Omatsu K. (2026). SJP-0132 clinical study investigators Efficacy and Safety of SJP-0132 in Patients with Dry Eye Disease: A Phase 2b Randomized, Double-Masked, Dose-Finding Study. Am. J. Ophthalmol..

[157] Stasi K., Alshare Q., Jain M., Wald M., Li Y. (2022). Topical Ocular TRPV1 Antagonist SAF312 (Libvatrep) Demonstrates Safety, Low Systemic Exposure, and No Anesthetic Effect in Healthy Participants. Transl. Vis. Sci. Technol..

[158] Thompson V., Moshirfar M., Clinch T., Scoper S., Linn S.H., McIntosh A., Li Y., Eaton M., Ferriere M., Stasi K. (2023). Topical Ocular TRPV1 Antagonist SAF312 (Libvatrep) for Postoperative Pain After Photorefractive Keratectomy. Transl. Vis. Sci. Technol..

[159] Kushnarev M., Pirvulescu I.P., Candido K.D., Knezevic N.N. (2020). Neuropathic Pain: Preclinical and Early Clinical Progress with Voltage-Gated Sodium Channel Blockers. Expert Opin. Investig. Drugs.

[160] Asiedu K. (2024). Neurophysiology of Corneal Neuropathic Pain and Emerging Pharmacotherapeutics. J. Neurosci. Res..

[161] Dormer A., Narayanan M., Schentag J., Achinko D., Norman E., Kerrigan J., Jay G., Heydorn W. (2023). A Review of the Therapeutic Targeting of SCN9A and Nav1.7 for Pain Relief in Current Human Clinical Trials. J. Pain Res..

[162] Wu Z., Lu D. (2026). Advances in the Discovery of Selective NaV1.8 Inhibitors for Pain Management. Eur. J. Med. Chem..

[163] Hijma H.J., Siebenga P.S., de Kam M.L., Groeneveld G.J. (2021). A Phase 1, Randomized, Double-Blind, Placebo-Controlled, Crossover Study to Evaluate the Pharmacodynamic Effects of VX-150, a Highly Selective NaV1.8 Inhibitor, in Healthy Male Adults. Pain Med..

[164] Price N., Namdari R., Neville J., Proctor K.J.W., Kaber S., Vest J., Fetell M., Malamut R., Sherrington R.P., Pimstone S.N. (2017). Safety and Efficacy of a Topical Sodium Channel Inhibitor (TV-45070) in Patients With Postherpetic Neuralgia (PHN). Clin. J. Pain.

[165] Nortey J., Smith D., Seitzman G.D., Gonzales J.A. (2022). Topical Therapeutic Options in Corneal Neuropathic Pain. Front. Pharmacol..

[166] McKay T.B., Seyed-Razavi Y., Ghezzi C.E., Dieckmann G., Nieland T.J.F., Cairns D.M., Pollard R.E., Hamrah P., Kaplan D.L. (2019). Corneal Pain and Experimental Model Development. Prog. Retin. Eye Res..

[167] Fabbretti E. (2013). ATP P2X3 Receptors and Neuronal Sensitization. Front. Cell. Neurosci..

[168] Merck Sharp & Dohme LLC A Phase 3, Randomized, Double-Blind, Placebo-Controlled, 12-Month Study to Evaluate the Efficacy and Safety of MK-7264 in Adult Participants with Chronic Cough (PN027); Clinicaltrials.gov, 2021. https://clinicaltrials.gov/study/NCT03449134.

[169] Ford A.P. (2012). In Pursuit of P2X3 Antagonists: Novel Therapeutics for Chronic Pain and Afferent Sensitization. Purinergic Signal..

[170] Belmonte C., Nichols J.J., Cox S.M., Brock J.A., Begley C.G., Bereiter D.A., Dartt D.A., Galor A., Hamrah P., Ivanusic J.J. (2017). TFOS DEWS II Pain and Sensation Report. Ocul. Surf..

[171] Kalangara J.P., Galor A., Levitt R.C., Felix E.R., Alegret R., Sarantopoulos C.D. (2016). Burning Eye Syndrome: Do Neuropathic Pain Mechanisms Underlie Chronic Dry Eye?. Pain Med..

[172] Galor A., Covington D., Levitt A.E., McManus K.T., Seiden B., Felix E.R., Kalangara J., Feuer W., Patin D.J., Martin E.R. (2016). Neuropathic Ocular Pain Due to Dry Eye Is Associated With Multiple Comorbid Chronic Pain Syndromes. J. Pain.

[173] Vázquez A., Martínez-Plaza E., Fernández I., Sobas E.M., González-García M.J., Enríquez-de-Salamanca A., Ortega E., López-Miguel A., Calonge M. (2022). Phenotypic Characterization of Patients Developing Chronic Dry Eye and Pain after Refractive Surgery: A Cross-Sectional Study. Ocul. Surf..

[174] Cheng J., Liu C., Yu M., Lee I.X.Y., Wang X., Hsu V.W.-T., Takahashi A., Mehta J.S., Zhou L., Tong L. (2025). Exploration of Imaging and Molecular Biomarkers for Differentiation of Neuropathic Corneal Pain from Dry Eye Syndrome. Ocul. Surf..

[175] Shetty R., Dua H.S., Tong L., Kundu G., Khamar P., Gorimanipalli B., D’Souza S. (2023). Role of in Vivo Confocal Microscopy in Dry Eye Disease and Eye Pain. Indian J. Ophthalmol..

[176] Ma L., Liu X., Liu Q., Jin S., Chang H., Liu H. (2022). The Roles of Transient Receptor Potential Ion Channels in Pathologies of Glaucoma. Front. Physiol..

[177] Wirta D.L., Senchyna M., Lewis A.E., Evans D.G., McLaurin E.B., Ousler G.W., Hollander D.A. (2022). A Randomized, Vehicle-Controlled, Phase 2b Study of Two Concentrations of the TRPM8 Receptor Agonist AR-15512 in the Treatment of Dry Eye Disease (COMET-1). Ocul. Surf..

[178] Garami A., Shimansky Y.P., Rumbus Z., Vizin R.C.L., Farkas N., Hegyi J., Szakacs Z., Solymar M., Csenkey A., Chiche D.A. (2020). Hyperthermia Induced by Transient Receptor Potential Vanilloid-1 (TRPV1) Antagonists in Human Clinical Trials: Insights from Mathematical Modeling and Meta-Analysis. Pharmacol. Ther..

[179] Esancy K., Dhaka A. (2024). Turning down the Body Heat: A Novel Mechanism for TRPV1 Antagonist-Induced Hyperthermia. Neuron.

[180] Garami A., Steiner A.A., Pakai E., Wanner S.P., Almeida M.C., Keringer P., Oliveira D.L., Nakamura K., Morrison S.F., Romanovsky A.A. (2023). The Neural Pathway of the Hyperthermic Response to Antagonists of the Transient Receptor Potential Vanilloid-1 Channel. Temperature.

[181] Gadziński P., Froelich A., Wojtyłko M., Białek A., Krysztofiak J., Osmałek T. (2022). Microneedle-Based Ocular Drug Delivery Systems—Recent Advances and Challenges. Beilstein J. Nanotechnol..

[182] Arabpour Z., Salehi M., An S., Moghtader A., Anwar K.N., Baharnoori S.M., Shah R.J., Abedi F., Djalilian A.R. (2024). Exploring Hydrogel Nanoparticle Systems for Enhanced Ocular Drug Delivery. Gels.

[183] Tawfik A., Pistilli M., Maguire M.G., Chen Y., Yu Y., Greiner J.V., Asbell P.A., Ying G. (2024). Association of Dry Eye Symptoms and Signs in Patients with Dry Eye Disease. Ophthalmic Epidemiol..

[184] Levitt A.E., Galor A., Chowdhury A.R., Felix E.R., Sarantopoulos C.D., Zhuang G.Y., Patin D., Maixner W., Smith S.B., Martin E.R. (2017). Evidence That Dry Eye Represents a Chronic Overlapping Pain Condition. Mol. Pain.

[185] Vehof J., Kozareva D., Hysi P.G., Harris J., Nessa A., Williams F.K., Bennett D.L.H., McMahon S.B., Fahy S.J., Direk K. (2013). Relationship Between Dry Eye Symptoms and Pain Sensitivity. JAMA Ophthalmol..

[186] Stapleton F., Alves M., Bunya V.Y., Jalbert I., Lekhanont K., Malet F., Na K.-S., Schaumberg D., Uchino M., Vehof J. (2017). TFOS DEWS II Epidemiology Report. Ocul. Surf..

[187] Jääskeläinen S.K., Teerijoki-Oksa T., Forssell H. (2005). Neurophysiologic and Quantitative Sensory Testing in the Diagnosis of Trigeminal Neuropathy and Neuropathic Pain. Pain.

[188] Rosenthal P., Borsook D. (2016). Ocular Neuropathic Pain. Br. J. Ophthalmol..

[189] Lazzarini D., Valerio A.L.G., Fregona I.A., Mocellin A., Midena E., Leonardi A. (2016). Neuropathic Ocular Pain Assessment. Investig. Ophthalmol. Vis. Sci..

[190] Eio E., Yu M., Liu C., Lee I.X.Y., Wong R.K.T., Wong J.H.F., Liu Y.-C. (2025). Evaluation of Corneal Sensitivity: Tools We Have. Diagnostics.

[191] Coco G., Buffon G., Taloni A., Giannaccare G. (2024). Recent Advances in Nanotechnology for the Treatment of Dry Eye Disease. Nanomaterials.

[192] Kannan R., Das S., Shetty R., Zhou L., Ghosh A., Deshpande V. (2023). Tear Proteomics in Dry Eye Disease. Indian J. Ophthalmol..

[193] Yang J.M., Li F., Liu Q., Rüedi M., Wei E.T., Lentsman M., Lee H.S., Choi W., Kim S.J., Yoon K.C. (2017). A Novel TRPM8 Agonist Relieves Dry Eye Discomfort. BMC Ophthalmol..

